# Pornographic consumption in young adult men and its relationship with stress and dissociative symptoms: A cross-sectional STROBE design

**DOI:** 10.1097/MD.0000000000048856

**Published:** 2026-05-15

**Authors:** Michal Privara, Petr Bob

**Affiliations:** a Center for Neuropsychiatric Research of Traumatic Stress, Department of Psychiatry and UHSL, First Faculty of Medicine, Charles University, Prague, Czech Republic.

**Keywords:** anxiety, dissociation, pornography, stress

## Abstract

Recent findings indicate that deficits in emotional regulation, stress and anxiety may influence lower sexual satisfaction, sexual dysfunctions and increased consumption of pornography. The objective of this research was to assess if traumatic stress and dissociative experiences from previous life manifested by typical symptoms will be related to increased levels of pornography consumption. In this observational study 1100 adult young men (mean age 27.55, age range 20–35, standard deviation = 5.21) were assessed for their pornography consumption behavior (sexual addiction screening test-revised, youth pornography addiction screening tool) and for their stress symptoms (trauma symptoms checklist-40), symptoms reflecting dissociative experiences (dissociative experiences scale), and symptoms of anxiety (self-rating anxiety scale). The results indicate that in these participants their pornography consumption experiences manifest significant positive Spearman correlations with stress symptoms (Spearman *r* = 0.47, *P* < .01), and mild correlations were also found for pornography consumption in its relationship to dissociative experiences and anxiety, and the results were also confirmed using Mann–Whitney test. The data indicate that the pornography consumption may be closely related to stress response, anxiety and defense mechanism related to dissociative experiences.

## 1. Introduction

According to current diagnostic and statistical manual of mental disorders-5 dependence on online pornography does not represent a separate syndrome but as some researchers and clinicians suggest it can be included as a part of a hypersexual disorder. According to the International Classification of Diseases (ICD-10), “excessive consumption of pornography” is diagnostically close to “excessive sexual urge” also denoted as hypersexuality (F52.7) representing enhanced but “a nondeviant manifestations of sexual behavior.” In addition to ICD-10, in ICD-11 disease classification the excessive pornography consumption is described in the context of “impulse control disorder.”^[1]^

In this context according to recent findings increased sexual interest and hypersexual behavior is related to deficits of interpersonal communication, which may implicate that deficits in intimate partnerships may be compensated by “object” related hypersexual behavior.^[2,3]^ In addition some findings suggest that hypersexuality can be a learned response to stressful experiences and may become a way to manage anxiety and negative emotions disturbances.^[3–6]^ Several findings also indicate that some individuals with this purpose to manage anxiety and negative emotions may use pornography and other forms of sexual stimulation.^[6–9]^

In addition, developmental findings also suggest that adults with adverse stressful and traumatic childhood experiences tend to experience sexual compulsions.^[10]^ Some findings also show that poor emotion regulation such as excessive use of suppression (and less frequent use of reappraisal) may influence lower sexual satisfaction, sexual dysfunctions and consumption of pornography.^[11,12]^ On the other hand recent literature does not include reported finding about the relationship of the pornography consumption with dissociative symptoms and symptoms of traumatic stress, and this study presents novel contribution with respect to the previous research on stress and hypersexual behavior.

Because increasing problematic pornography use in male population potentially may have negative consequences for mental health we have tested a hypothesis if traumatic stress and dissociative experiences from previous life manifested by typical symptoms will be related to increased levels of pornography consumption in 1100 male consecutive participants selected consecutively using advertising from general population.

## 2. Participants and methods

### 2.1. Participants

In the present study 1100 adult young men were assessed for their symptoms of stress, dissociation, anxiety and their pornography related behavior. The sample included consecutive participants obtained by advertising from general population which mainly included University students (mean age 27.55, age range 20 to 35, standard deviation [SD] = 5.21). The participants had predominantly high school education 15.31 (SD = 4.41) years. Other inclusion or exclusion criteria were not applied. All the participants gave written informed consent and the study research and ethical criteria were approved by the Charles University committee for Medical Psychology and Psychopathology (MedPsych MPRP20-06). The period for data acquisition was from January to May 2022. All used methods of psychological investigation were performed in accordance with the relevant guidelines and regulations.

### 2.2. Psychometric measures

For investigation of childhood traumas, trauma symptoms checklist (TSC)-40^[13]^ was used. The TSC-40 is a 40-item self-reported questionnaire done on a 4-point Likert scale. TSC-40 evaluates symptoms in adults associated with childhood or adult traumatic experiences and measures aspects of posttraumatic stress and other symptom clusters found in some traumatized individuals (Cronbach alpha 0.91, test-retest reliability after week 0.88).

Psychic dissociative symptoms were assessed by dissociative experiences scale (DES).^[14]^ DES represents 28 items self-reported questionnaire examining main dissociative phenomena such as absorption, amnesia, depersonalization, derealization, reality distortion, and others. Subjects indicate a degree of their experience on the continuum from 0 to 100% (Cronbach alpha 0.92, test-retest reliability after week 0.91).

Levels of anxiety symptoms were assessed using the Zung self-rating anxiety scale (SAS) (Cronbach alpha 0.89, test-retest reliability after week 0.85).^[15]^ The SAS is 20-item self-reporting questionnaire focused on the most common general anxiety symptoms. Each question is scored on 4-point Likert scale from 1 to 4.

Related potential sexual problems and addiction were assessed by sexual addiction screening test-revised (SAST-R). The SAST-R is a 45-item screener for detecting potential cases of sexual addiction. The SAST-R consists of the 20-item Core scale measuring the general construct of sexual addiction; 4 subscales measuring constituent components: preoccupation, loss of control, relationship disturbance and affective disturbance. Internet scale, measuring internet-related sexual activity. Cronbach alpha for the Core and SAST-R are 0.86 and 0.79.^[16]^

Symptoms of problematic pornography use were measured by youth pornography addiction screening tool (YPAST). This scale includes 25 items scored on a 5-point Likert scale ranging from 0 to 4 (options of never, very rarely, rarely, sometimes, and often). This questionnaire measures the degree of addiction to virtual pornography, with high scores indicating the addiction of the subject to virtual pornography. This questionnaire is suitable for the age group of 18 to 40 years. Its construct validity using exploratory factor analysis is 0.48 Cronbach alpha is 0.79.^[17,18]^

### 2.3. Statistical analysis

Statistical evaluation of psychometric measures included means and SDs, and because dissociation does not have normal distribution we have used nonparametric Spearman correlation coefficients. In addition with the purpose to compare subgroups S1 with TSC-40 scores lower than median and S2 with TSC-40 higher than median nonparametric Mann-Whitney test was used. All the methods of statistical evaluation were performed using the software package Statistica version 11 (StatSoft, Tulsa). Because the data do not have normal distribution we have used mainly nonparametric statistical analysis (Spearman correlations coefficients, Mann-Whitney test). The main advantage to use nonparametric analysis is its very conservative approach to outliers and leverage points, which in the case of using parametric correlations or regression analysis may create false results and increase risk of inappropriate rejection of the null hypothesis. In addition before the data collection we have used power analysis to estimate the number of participants with respect to a possibility to obtain significant results in specific conditions of the research study related to co-occurrence of stress related variables and pornography consumption and their potential relationships.

## 3. Results

**Figure 1.**
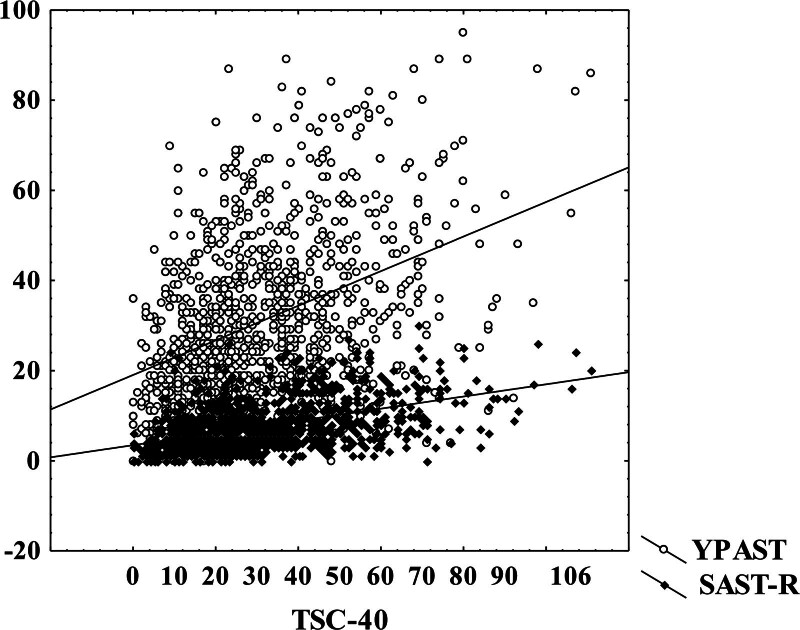
Relationships of pornography consumption measured by YPAST and SAST-R with stress symptoms measured by TSC-40. Spearman correlation between TSC-40 and YPAST is *r* = 0.40, *P* < .01, and between TSC-40 and SAST-R is *r* = 0.47, *P* < .01. SAST-R = sexual addiction screening test-revised, TSC = trauma symptoms checklist, YPAST = youth pornography addiction screening tool.

Results indicate that in the participants who manifest higher levels of stress symptoms than median (S1 subgroup) is pornography consumption and other sexual problems higher than in participants who experience lower levels of stress as indicated by Mann-Whitney test (Table 1) and also by correlation analysis and power analysis to estimate the smallest sample size needed for the assessment. Stress symptoms measured by TSC-40 manifest significant correlations with pornography consumption measured by YPAST (Spearman *r* = 0.40, *P* < .01) and SAST-R (Spearman *r* = 0.47, *P* < .01) (Fig. 1). Mild correlations were found between dissociative symptoms measured by DES and pornography consumption measured by YPAST (Spearman *r* = 0.21, *P* < .01), and SAST-R (Spearman *r* = 0.29, *P* < .01), and also between anxiety symptoms measured by SAS and pornography consumption measured by YPAST (Spearman *r* = 0.23, *P* < .01), and SAST-R (Spearman *r* = 0.27, *P* < .01).

**Table 1:** Statistical analysis describing descriptive results of psychometric measures and statistical differences using Mann-Whitney test between the subgroups S1 and S2 (S1 with TSC-40 scores lower than median and S2 with TSC-40 higher than median) with *P* < .01 for the all statistical comparisons.

	Mean	SD	Mean-S1	SD-S1	Mean-S2	SD-S2	*U*	*Z*
YPAST	30.95	17.45	25.41	14.11	36.73	18.69	95,405.50	−10.59
SAST-R	7.64	5.36	5.71	4.34	9.65	5.58	85,354.50	−12.50
TSC-40	31.05	18.94	16.53	7.20	46.17	15.17	679.50	−28.58
SAS	36.25	6.63	33.14	4.38	39.50	7.01	64,536.50	−16.45
DES	376.13	332.50	245.79	217.51	511.78	374.76	70,299.50	−15.36

DES = dissociative experiences scale, S1 = subgroup 1, S2 = subgroup 2, SAS = self-rating anxiety scale, SAST-R = sexual addiction screening test-revised, SD = standard deviation, TSC = trauma symptoms checklist, YPAST = youth pornography addiction screening tool.

## 4. Discussion

The results are in agreement with the hypothesis tested in this research that in the participants who manifest higher levels of stress symptoms is pornography consumption and other related sexual problems higher than in participants who experience lower levels of stress. According to our research review of the recent literature there is just 1 reported study indicating a relationship between pornography use and traumatic stress symptoms based on assessment of a traumatic history, which is the study by Aaron,^[10]^ who reported that adults with adverse stressful childhood experiences tend to experience sexual compulsions. Findings of this study are also in agreement with previously published studies indicating that increased pornography consumption is frequently linked to stressful life events and anxiety.^[1–6]^

Although the correlations observed were relatively low, it is essential to highlight that these correlations might have potential implications for understanding the relationship between stress, dissociative experiences, and pornography consumption. These correlations indicate “bidirectional” causality relationship between assessed variables, which suggests that simple causality of stress related variables and pornography consumption is not possible. In this context bidirectional causality means previous stress and traumatic experiences negatively influence various skills which are necessary to create interpersonal and sexual relationships and using pornography may help to decrease internal psychological tension. In addition recently experienced stressful experience also may negatively affect confidence in interpersonal relationships and pornography may substitute this internal gap. On the other hand pornography provides experience that may create ambivalent feelings and may be frustrating and satisfying at the same time which may negatively influence mental integrity and indirectly influence dissociative symptoms and in this context “dissociative tendencies” may be conceptualized as mediators or moderators of this relationship stress and pornographic experiences. This relationship is also in agreement with some recent findings indicating that excessive pornography consumption may have negative influence on sexual development in childhood and adolescence and on the other hand also recently experienced stress may influence increased pornography consumption.^[3,4,19]^

Limitation of this study with respect to validity of this research for the general population is mainly linked to the critical reflection of cultural and contextual factors because the sample mainly included Czech University students, who represents very homogenous sample with very low cultural or ethnical diversity. In this sample we did not asses other psychopathological symptoms and not applied other exclusion or inclusion criteria. The goal for this research was not to exclude participants with possible psychopathological symptoms or mental diseases in this sample. The opposite was true and the goal of this research was to find a relationship between stress experiences and pornography consumption patterns.

Limitation of this research with respect to this sample homogeneity were also other possible statistical analyses such as regression models that could provide more detailed information about potential confounders such as age, education level, comorbid anxiety or other psychopathological symptoms. With respect to this limitation related to the sample homogeneity and not “normal distribution” of the data we did not use these analyses for potential confounders to statistically confirm what “we already knew” based on the data acquisition criteria.

In this context also sociocultural attitudes toward pornography and masculinity might influence both self-reporting and consumption patterns, which affect the generalizability of results. These mainly include traditional European conservative views and prototypes of masculinity and feminity and “unconsciously” experienced Christian heritage which may influence idealistic imagination about relationships between men and women and in many cases strong guilty feelings related to the consumption of pornography. These guilty feelings may cause ambivalent feelings mentioned above which may influence manifestation of dissociative symptoms.

In this context of the limitations mentioned above also the statistically significant correlations and their clinical significance are limited with respect to the culturally informed clinical practice where ethnical diversity may play a significant role. Nevertheless within “European cultural context” anywhere around the world these results may provide useful information for psychological assessment and intervention strategies in mental health or sex therapy settings.

Although excessive pornography consumption still does not represent a diagnostic entity it may have mental health consequences.^[20–23]^ Nevertheless in agreement with ICD-11 disease classification the excessive pornography consumption may be diagnosed as “impulse control disorder” and defined as a failure to control intense sexual impulses or compulsions, which leads to repetitive sexual behavior and causes significant suffering or significant disruptions in personal, family and social life.^[1,23]^

In summary recent findings, including the novel results of this study, suggest that although “pornography addiction” still it is not recognized as a disorder it might represent a developmental and etiological factor with negative influences on mental health. Mainly some findings suggest that the viewing pornography in childhood and adolescence may negatively influence forming of sexual identity and relationships, mainly due to various “unrealistic” gender stereotypes presented in pornographic roles and relations, which are also characterized by physical perfectionism and narcissistic body image^[1,19,24]^ that may have negative effects on personality development and mental integrity.

Basic limitation regarding causal interpretation of these findings is potential “bi-directional causality,” because the already distressed persons have higher probability to manifest pornography related addictive behavior or problematic pornography use, and on the other hand it is possible that frequently used pornography may cause internal distress and increase levels of stress related psychopathological symptom. Another limitation because of the very large sample was the inability to provide detailed screening of occurrence of psychiatric disorders. Nevertheless the sample in majority of cases included university students, which implicates that from the epidemiological perspective manifestations of mental diseases in the sample should not be higher than in the representative sample from the general population. From the perspective to find more general findings more detailed information and analysis are needed. For example, future studies could include multivariate and multivariable nonparametric data analyses to find other potential confounders of pornographic consumption such as social learning, peer factors, availability of porn sites and other important variables.

These issues might be resolved in the longitudinal study design and further research is warranted. From the clinical perspective further research that would enable to find more details about the link between stress and pornography consumption could help clinicians to recognize underlying emotional dysregulation related to trauma and stress experiences in individuals exhibiting problematic pornography use and could help to find appropriate intervention strategies.

## Acknowledgments

This research was supported by Charles University project Cooperation SVV

## Author Contribution:

**Conceptualization:** Petr Bob.

**Data curation:** Michal Privara.

**Formal analysis:** Petr Bob.

**Funding acquisition:** Petr Bob.

**Investigation:** Michal Privara, Petr Bob.

**Methodology:** Michal Privara, Petr Bob.

**Project administration:** Michal Privara, Petr Bob.

**Software:** Petr Bob.

**Validation:** Michal Privara, Petr Bob.

**Writing – original draft:** Michal Privara, Petr Bob.

**Writing – review & editing:** Michal Privara, Petr Bob.
